# Utilizing Untargeted Lipidomics Technology to Elucidate Differences in Lipid Compositions Among Sensitive Dry, Sensitive Oily and Healthy Skin Types

**DOI:** 10.3390/metabo15050292

**Published:** 2025-04-26

**Authors:** Agui Xie, Xingjiang Zhang, Qing Huang, Jianxin Wu

**Affiliations:** Skin Health and Cosmetic Development & Evaluation Laboratory, China Pharmaceutical University, Nanjing 211198, China; xieagui@stu.cpu.edu.cn (A.X.); 3122024136@stu.cpu.edu.cn (X.Z.)

**Keywords:** sensitive skin, lipidomics, skin barrier dysfunction, sphingolipids

## Abstract

**Background:** Sensitive skin exhibits impaired skin barrier function. The lipid composition of the skin, a pivotal element within the stratum corneum’s “brick-and-mortar” structure, plays a dual role: it is integral to cell differentiation processes and serves as a vital nutrient reservoir for cutaneous microbiota, thereby influencing the skin’s microecological balance. There is a notable research gap concerning the comparative analysis of physiological parameters and lipid profiles among individuals with sensitive dry skin (SDS), sensitive oily skin (SOS), and healthy skin (HS). **Methods:** A total of 95 females (18–25 years) were grouped: SDS (*n* = 32), SOS (*n* = 31), and HS (*n* = 32). Stratum corneum water content, oil content, and TEWL were measured. Lipids from sebaceous glands and stratum corneum (tape-stripping) underwent UPLC-QTOF-MS analysis. Differential lipids were identified via OPLS-DA, volcano plots, and LMSD. **Results:** In terms of physiological indicators, notable disparities emerged in oil content and stratum corneum water content between the SOS and both the HS and the SDS. Sensitive skin, whether dry or oily, displayed a higher transepidermal water loss (TEWL) value than healthy skin, reflecting a declined state of skin barrier function. Regarding the sebum samples, the relative percentages of sphingolipids (SP) and glycerophospholipids (GP) were significantly higher in SDS. Regarding the stratum corneum samples, the percentages of SP in SDS were significantly higher. **Conclusions:** This study, for the first time, conducted a comprehensive analysis of the skin’s physiological properties, lipidomics of sebum, and stratum corneum lipids among groups with SDS, SOS, and HS. These observations indicate a profound association between skin barrier dysfunction in SDS individuals and, in particular, sphingolipids (SP).

## 1. Introduction

Sensitive skin represents a condition marked by hyperreactivity, reduced tolerance, and a heightened propensity to allergic reactions, primarily affecting the facial area, with cheeks and cheekbones being the most prevalent sites [1,2]. When exposed to physical, chemical, or psychological stimuli, sensitive skin exhibited subjective symptoms such as burning, tingling sensations, itchiness, and a feeling of tightness, in contrast to healthy skin [3]. Occasionally, these reactions were accompanied by objective manifestations like erythema, scaling, and capillary dilation [4].

Numerous factors were found to contribute to the development of sensitive skin, with skin barrier dysfunction emerging as a primary contributing factor [5,6]. The skin barrier was described as a “bricks and mortar” structure, in which keratinocytes and their internal proteins served as the “bricks” and intercellular lipids functioned as the “mortar”, collectively sustaining the structural integrity and functional stability of the skin barrier [7,8]. This barrier was crucial for retaining skin moisture and nutrients while effectively blocking the penetration of external harmful substances and microorganisms [9,10,11].

Individuals with sensitive skin commonly exhibited a decrease in oil content and stratum corneum water content and an increase in transdermal water loss (TEWL), which was undoubtedly direct evidence of impaired skin barrier function [12].

Epidermal differentiation was a key process in the formation of the skin barrier, involving the transition of cells from a proliferative state to terminal differentiation, culminating in the creation of a protective stratum corneum [13]. During this developmental journey, lipids, e.g., sphingolipids (SP), served as the core components of epidermal differentiation, not only forming the physical structure of the barrier but also playing a pivotal role in the regulation of cell signaling pathways. Lipids, based on their origin and composition, were classified into two primary groups: sebaceous gland lipids and intercellular lipids [14]. Sebaceous gland lipids, constituting a dominant proportion (75–90%), abounded in crucial components like squalene, triglycerides, cholesterol, and fatty acids. Conversely, intercellular lipids, comprising 10–25% of the total, had ceramides as their signature component and were indispensable for the stabilization of the skin barrier [15,16].

The process of epidermal differentiation was intricately linked to lipid synthesis, commencing from keratinocyte cells in the basal layer and progressing through the stratum spinosum and stratum granulosum, before ultimately yielding stratum corneum cells. The synthesis and assembly of lipids during this process were of paramount importance, particularly for intercellular lipids, whose synthesis and distribution were decisive in maintaining the integrity of the skin barrier. Ceramide, as a key constituent of intercellular lipids, not only fostered the tight junctions between keratinocytes and enhanced the barrier function of the skin but also played a central role in preserving the structural integrity of the stratum corneum. Moreover, ceramide participated in the differentiation and repair processes of the skin, exerting a profound impact on the skin’s health and disease state [17].

In addition to ceramides, the skin barrier depends on a coordinated lipid network where distinct classes execute specialized roles: glycerophospholipids (GP) maintain membrane integrity through amphipathic bilayers, while glycerolipids (GL) regulate sebum fluidity [18]. Polyketides (PK) and sterol lipids (ST) exhibit anti-inflammatory and immune-regulatory effects [19,20,21]. Fatty acyls (FA), particularly long-chain types, reduce water loss and help block external irritants, synergizing with sphingolipids (SP)—particularly ceramides constituting >50% of stratum corneum lipids—to form liquid–crystalline matrices. Prenol lipids (PR) mediate cellular signaling through isoprenoid derivatives [22]. Together, these lipid classes form a well-organized system that supports both the physical and functional aspects of the skin barrier.

In this context, lipidomics emerges as a cutting-edge analytical technique, offering an indispensable tool to unveil the intricate lipid dynamics underlying skin health and disease conditions. Its distinctive prowess lies in the large-scale interrogation of lipids and the comprehensive assessment of their composition and functionality within biological systems [23,24]. In this study, an untargeted lipidomics approach was used, which can comprehensively detect and identify all lipid species in a sample without the need for predefined target molecules and further classify and analyze the detected lipid peaks in detail through advanced computational sequencing and multivariate pattern recognition techniques [25]. Untargeted lipidomics has demonstrated extraordinary capabilities in the discovery of novel lipids and screening of differential lipids, opening up avenues for finding lipid biomarker candidates [26,27].

Alongside booming social and economic development, people’s living standards are improving, and the pursuit of health and beauty is becoming increasingly strong. This pursuit is not only limited to physical health, but also extends to the level of skin health. Most previous studies have focused primarily on macroscopic lipid changes in the epidermis, ignoring the differences between sebaceous gland metabolism and the lipid structure of the stratum corneum. In addition, few studies have explored the differences in physiologic indices between sensitive and healthy skin, as well as the apparent differences in lipid composition within the sebaceous glands and stratum corneum intercellular between these two skin types. To further explore these areas, a group of college students aged 18–25 years was selected for this study. The primary objective was to examine the differences in facial physiological indices and lipid composition in sebaceous glands and the intercellular spaces of the stratum corneum between sensitive and healthy skin types in females. The study aimed to elucidate the variations in lipid composition and their distribution patterns by integrating physiological index assessments with untargeted lipidomics techniques. Furthermore, the study aimed to identify metabolizable lipids that exhibit significant differences, thereby providing a fundamental basis for the management of skin health and the development of targeted skincare products.

## 2. Materials and Methods

### 2.1. Study Participants

Participants in this study were recruited from Zhengzhou University and classified into three groups: sensitive oily skin (SOS), sensitive dry skin (SDS), and healthy skin (HS). The inclusion criteria were as follows: (1) female college students aged between 18 and 25 years old; (2) SOS group: individuals exhibiting positive lactic acid tingling, with an oil content of at least 120 μg/cm^2^, and a Corneometer value exceeding 45 a.u. at the intersection of the eye corner and mouth corner [28]; (3) SDS group: individuals demonstrating positive lactic acid tingling, a Corneometer value of 45 a.u. or below at the aforementioned intersection, and an oil content below 120 μg/cm^2^; (4) HS group: individuals without positive lactic acid tingling, an oil content below 120 μg/cm^2^, and a Corneometer value exceeding 45 a.u. at the aforementioned intersection. The exclusion criteria were as follows: (1) subjects who were pregnant, breastfeeding, or planning to become pregnant; and those (2) suffering from severe systemic diseases. A total of 95 study subjects were finally enrolled, including 32 SDS, 32 HS, and 31 SOS. This study was approved by the Life Sciences Ethics Review Committee of Zhengzhou University. Before the experiment began, all volunteers were informed of the purpose of the experiment and signed an informed consent form.

### 2.2. Sample Collection and Preparation

#### 2.2.1. Test Environmental Conditions

Throughout the instrumental testing session, the test should be controlled in an environment with a temperature of 20 ± 2 °C, a relative humidity of 50 ± 10% RH, and a color temperature of 6500 K.

#### 2.2.2. Chemical Reagents and Instruments

Filter paper disks with a diameter of 0.8 cm; pressure stick (225 g/cm^2^); sebum sampling tape (Sebutape^®^-S100, Clinical and Derm, Dallas, TX, USA); stratum corneum sampling tape (D-Squame^®^-D100, Clinical and Derm, Dallas, TX, USA); 4 mL Eppendorf (EP) tubes; analytical balance with a precision of 0.1 mg; acetonitrile (ACN), methanol (MT), isopropanol (IPA), formic acid, and ammonium formate were of LC grade. MPA 580 (Courage & Khazaka, Köln, Germany), equipped with Sebumeter^®^ SM815, Corneometer^®^ CM825, Tewameter^®^ TM300, and Colorimeter^®^ CL400 probes; Vortex Mixers (Scilogex, Rocky Hill, CT, USA), Mass Spectrometer (UPLC-VION QTOF MS, Waters, Milford, MA, USA), nitrogen evaporator (ANPEL, Shanghai, China).

#### 2.2.3. Physiological Indicator Measurements

Volunteers were required to clean their faces with water at 21:00 on the previous day, after which re-cleansing or the use of any skincare/makeup products was prohibited until the start of the experiment. Upon arrival, the subjects first cleansed their faces using water. They were then placed in an environment with constant temperature and humidity for 30 min to stabilize their skin condition. Then, Sebumeter, Corneometer, and Tewameter were used to measure the stratum corneum water content, oil content, and TEWL, respectively, and the data obtained were analyzed and recorded.

#### 2.2.4. Skin Lipid Sampling

After completing the physiological index measurements, the lipid peeling tape method was employed to collect sebaceous gland lipids and stratum corneum intercellular lipids from the volunteers’ faces [29]. (1) Initially, the grease tape was attached to the sebaceous gland lipid sampling site, and the pressure bar was utilized to press evenly for 3 s to ensure that the tape was tightly adhered to the skin. After 30 min, the tape was carefully removed and placed in a 4 mL EP tube. (2) After the sebaceous gland lipid sampling, the stratum corneum intercellular lipids were sampled. The stratum corneum tape was applied to the same sampling site, pressed with a pressure bar for 15 s, quickly removed, and placed into the corresponding 4 mL EP tube. (3) The tape and the EP tube were weighed accurately after sampling, and the samples were stored in a refrigerator at −80 °C immediately after completion for subsequent analysis.

### 2.3. Sample Processing

After removing the samples from the −80 °C refrigerator, 2 mL of methanol was quickly added to the EP tube and vortexed to mix. After incubation at room temperature with shaking for one hour, the sampling strip was removed and the extract was retained. Next, the extract of each sample was blown dry with nitrogen. Subsequently, 200 µL of a solvent mixture of acetonitrile, isopropanol, and water in a ratio of 65:30:5 was added and vortexed for thirty seconds to thoroughly re-dissolve the samples. Then, the samples were transferred to the sample vials and prepared for assay. Prior to ultra-performance liquid chromatography quadrupole time of flight mass spectrometry (UPLC-QTOF-MS) analysis, a mixture of all samples was prepared as a quality control (QC) sample, which was prepared from a mixture of sample extracts, and, during instrumental analysis, one QC sample was inserted in each of the eight assayed analytical samples in order to monitor the reproducibility and stability of the instrument during the analysis. Chromatographic analyses were performed using an ACQUITY UPLC CSH C18 column (1.7 µm 2.1 × 100 mm) with a flow rate set at 0.3 mL/min and an injection volume of 3 µL, and the column temperature was maintained at 50.0 °C. The mobile phase A consisted of 60% acetonitrile, 40% water, 10 mM ammonium formate, and 0.1% formic acid, and the mobile phase B consisted of 10% acetonitrile, 90% isopropanol, and 0.1% formic acid. The gradient elution conditions of mobile phases A and B as well as the mass spectrometry conditions are shown in Table S1 and Table S2, respectively [30].

### 2.4. Statistical Analysis

In this study, positive and negative ion modes were employed in the analysis of lipidomics samples to comprehensively capture different types of lipid molecules. MS2 fragment ions and retention time were used for identification, and the mass accuracy tolerance was set to 5 ppm for metabolite searches. Firstly, the raw data collected by the instrument were imported into Waters Progenesis QI V2.4 software for pre-processing such as peak alignment, peak extraction, and mass calibration. Subsequently, the abundance of compounds was normalized and molecular features were extracted. Compound identification was performed by matching the molecular formulae and fragment ion information of detected compounds against the public databases HMDB (https://www.hmdb.ca/ accessed on 22 July 2024) and LMSD (https://www.lipidmaps.org/databases/lmsd/overview accessed on 24 July 2024). Compounds were considered confidently identified based on their accurate mass-to-charge ratio (m/z) values, fragment ion patterns, and database matching scores. Additionally, to ensure reliability and utility, lipids detected in at least a third of all samples were retained. Missing values were imputed using the k-nearest neighbor algorithm (k = 5) implemented in Python 3.6. The processed data were imported into Metaboanalyst 6.0 website (https://new.metaboanalyst.ca/ModuleView.xhtml accessed on 30 July 2024), and models were constructed using orthogonal projections to latent structures discriminant analysis (OPLS-DA) to reveal differences between groups. The model was considered valid if the model R^2^X > 0.20, R^2^Y > 0.20, and Q^2^ > 0.00. The model fit was judged using the 1000 permutation test, and the model was considered meaningful if the *p* values for both R^2^Y and Q^2^ were less than 0.05. Using the R packages (R version 4.4.2; ggplot2, https://ggplot2.tidyverse.org; cowplot 1.1.3, https://wilkelab.org/cowplot/ accessed on 30 July 2024), the fold change in metabolite differences (FC) > 2 and *t*-test (*p*) < 0.05 were added to the initial screening criteria and visualized using volcano plots. If the number of metabolites was large, the ≤50 most representative differential metabolites were selected according to FC size.

## 3. Results

### 3.1. Differences in Skin Physiological Indicators of SDS, SOS, and HS Groups

As shown in Table 1, for the comparison of skin physiological parameters (oil content, stratum corneum water content, and TEWL) between the SDS, SOS, and HS groups, the results showed that no statistically significant differences were observed between the SDS and HS groups in all three assessed metrics (*p* > 0.05). Although there was no significant difference, it can be seen that the oil content (38.22 ± 26.18) and stratum corneum water content (40.10 ± 9.32) of the SDS group were lower than that of the HS group (45.26 ± 24.38 and 57.16 ± 7.96), and the TEWL value (10.88 ± 2.79) was higher than that of the HS group (9.26 ± 2.48).

Conversely, comparing the SOS group with the HS group, significant differences were found in oil content and stratum corneum water content (*p* < 0.05). Specifically, the oil content of the SOS group (105.23 ± 52.93) was significantly higher than that of the HS group (45.26 ± 24.38), whereas the water content of the HS group (57.16 ± 7.96) was slightly higher than that of the SOS group (52.69 ± 12.65). The TEWL values remained statistically indistinguishable between these two groups (*p* > 0.05). However, TEWL was higher in the SOS group (10.13 ± 2.28) than in the HS group (9.26 ± 2.48).

Further analysis of the differences between the SDS and SOS groups revealed that significant differences also existed in oil content and stratum corneum water content (*p* < 0.05). Specifically, the SOS group had significantly higher oil content (105.23 ± 52.93) and stratum corneum water content (52.69 ± 12.65) than the SDS group (38.22 ± 26.18 and 40.10 ± 9.32, respectively). Despite the absence of a statistically significant difference in TEWL values between the two groups (*p* > 0.05), it is noteworthy that the SDS group exhibited a marginally elevated TEWL (10.88 ± 2.79) compared to the SOS group (10.13 ± 2.28) (the relevant raw data and box plots are shown in Table S3 and Figure S1 in the Supplementary Materials).

### 3.2. Comprehensive Pairwise Comparative Lipidomic Analysis of Sebaceous Glands Across Three Distinct Skin Types

#### 3.2.1. Comparison of SDS and HS Groups in Sebaceous Glands

**Figure 1:**
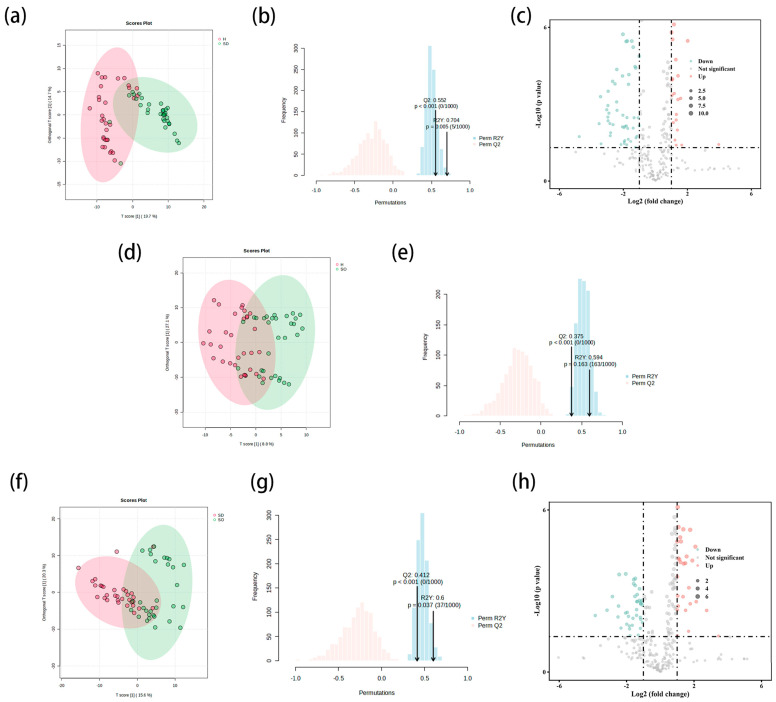
Differences in sebaceous gland lipids between the SDS and HS groups. (**a**–**c**) The score plot, permutation plot, and volcano plot for differential lipids in the sebaceous glands of the SDS and HS groups; (**d**,**e**) the scores plot and permutation plot for differential lipids in the sebaceous glands of the SOS and HS groups; (**f**–**h**) the score plot, permutation plot, and volcano plot for differential lipids in the sebaceous glands of the SDS and SOS groups. SD: sensitive dry; SO: sensitive oily; H: healthy.

The lipid data of the samples were subjected to OPLS-DA using Metaboanalyst 6.0, and the score plots were utilized to observe whether there was a significant difference between the two groups. As depicted in Figure 1a, a good separation of the lipids existed between the two groups. Subsequent validation of the model, complemented by a 1000-permutation test, demonstrated that the SDS and HS groups were significantly differentiated in the distribution of lipid material (R^2^Y = 0.704, Q^2^ = 0.552). Moreover, the *p* values for both R^2^Y and Q^2^ were less than 0.05 (Figure 1b). Therefore, the model was considered to be meaningful. Screening and analysis of differential lipid substances in the SDS and HS groups by volcano plot (Figure 1c) identified 297 lipid substances. Using *p* < 0.05 and fold change >2 as the selection criterion, 101 differentiated lipid constituents were further screened (Table 2 shows only the first 50 substances with large FC values), which were categorized into seven classes. Compared to the HS group, the expression of different kinds of lipids was decreased in the SDS group. Among them, glycerophospholipids (GP), glycerolipids (GL), polyketides (PK), sphingolipids (SP), sterol lipids (ST), and fatty acyls (FA) were relatively high in the HS group, whereas the content of prenol lipids (PR) did not have a significant impact.

#### 3.2.2. Comparison of SOS and HS Groups in Sebaceous Glands

Differential lipid matter was visualized using OPLS-DA for the SOS and HS groups (Figure 1d), which did not show a significant trend of separation between the two groups. A 1000-permutation test of the model revealed a lack of clear differentiation in the distribution of lipid substances (R^2^Y = 0.595, Q^2^ = 0.375), with a *p* value of greater than 0.05 for R^2^Y and a *p* value of less than 0.001 for Q^2^ (Figure 1e), and the model was therefore not considered to be meaningful.

#### 3.2.3. Comparison of SDS and SOS Groups in Sebaceous Glands

Upon employing OPLS-DA to investigate the lipid composition disparities between the SDS and SOS groups, a distinct segregation between the two cohorts was evident (Figure 1f), signifying substantial variations in their lipid profiles. Following rigorous validation through 1000 permutation tests, the model exhibited robust discriminatory power (R^2^Y = 0.6, Q^2^ = 0.412), with *p* values for both R^2^Y and Q^2^ falling beneath the 0.05 threshold for statistical significance (Figure 1g), thereby reinforcing the model’s credibility. A volcano plot (Figure 1h) was utilized for the screening and analysis of differential lipid substances, ultimately identifying a total of 297 distinct lipid entities. Applying stringent screening criteria (*p* < 0.05, fold change > 2), 69 highly significant lipid components were further isolated and classified into seven principal categories (Table 3 shows only the first 50 substances with large FC values). The results of the study showed that SP was relatively high in SDS types, while lipid components such as GP, GL, PK, PR, ST, and FA exhibited high levels in SOS.

### 3.3. Comprehensive Pairwise Comparative Lipidomic Analysis of Stratum Corneum Intercellular Across Three Distinct Skin Types

#### 3.3.1. Comparison of SDS and HS Groups in Stratum Corneum

**Figure 2:**
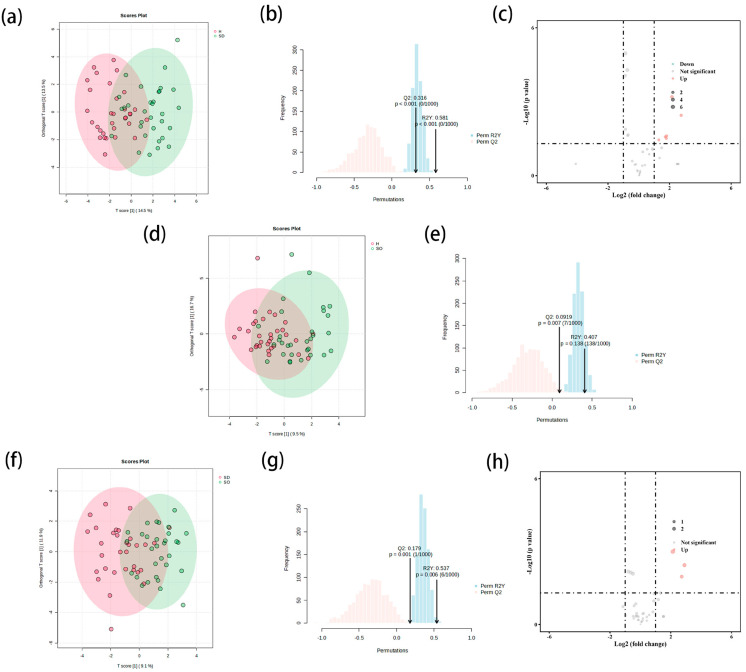
Intercellular lipid differences in the stratum corneum of the SDS and HS groups. (**a**–**c**) The scores plot, permutation plot, and volcano plot for differential lipids in the stratum corneum of the SDS and HS groups; (**d**,**e**) the scores plot and permutation plot for differential lipids in the stratum corneum of the SOS and HS groups; (**f**–**h**) the score plot, permutation plot, and volcano plot for differential lipids in the stratum corneum of the SDS and SOS groups. SD: sensitive dry; SO: sensitive oily; H: healthy.

As shown in Figure 2a, by using the same method, a significant difference was observed in the distribution of lipid materials between the SDS and HS groups. The model exhibited an explanatory rate (R^2^Y) of 0.581 and a predictive rate (Q^2^) of 0.316. To validate the robustness of the model, 1000 permutation tests were conducted. It was revealed that the *p* values of the permutation tests for both R^2^Y and Q^2^ were less than 0.05 (Figure 2b). This result indicated that the model was statistically significant and thereby supported its validity in differentiating between SDS and HS types. Afterwards, the lipids in these two groups were screened and analyzed using volcano diagrams (Figure 2c). A total of 38 lipid components were screened out. Among them, seven different lipid components were further screened based on the criteria of *p* < 0.05 and fold change > 2, and classified into four categories. Among them, PK was relatively high in the HS group, and GL, SP, and FA were relatively high in the SDS group (Table 4).

#### 3.3.2. Comparison of SOS and HS Groups in Stratum Corneum

As shown in Figure 2d, no significant separation pattern of the different lipid materials between the two groups was observed. The explanatory power (R^2^Y = 0.407) and predictive power (Q^2^ = 0.092) of the model indicated that the difference in lipid composition between the two groups of samples was not significant. Additionally, a 1000-permutation test revealed that while the *p* value for R^2^Y failed to reach the statistically significant level (*p* > 0.05), the *p* value for Q^2^ was less than 0.05 (Figure 2e), and the model was therefore not considered to be meaningful.

#### 3.3.3. Comparison of SDS and SOS Groups in Stratum Corneum

As shown in Figure 2f, the two groups of samples exhibited a significant separation trend in the distribution of lipid substances. Validated by the 1000-permutation test, the *p* values of the model’s R^2^Y value (0.537) and Q^2^ value (0.180) were significantly lower than the critical value of 0.05 (Figure 2g), thus confirming that the model was statistically significant. On this basis, the volcano diagram was employed to screen and identify the differential lipid substances between SDS and SOS types. Eventually, three lipid components with significantly high levels in SP were identified (Figure 2h), which were relatively high in SDS types (Table 5).

### 3.4. Analysis of the Lipids in the Sebaceous Glands of SDS, SOS and HS

**Figure 3:**
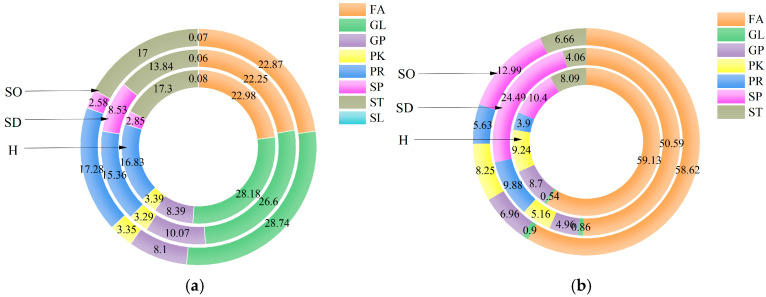
(**a**) Eight lipids of the sebaceous glands in the SDS, SOS, and HS groups; (**b**) seven lipids of the stratum corneum in the SDS, SOS, and HS groups. SD: sensitive dry; SO: sensitive oily; H: healthy.

After comprehensive analysis of sebaceous gland lipids from the SDS, SOS, and HS groups, a total of 297 lipid components were identified. These lipids were categorized into eight major groups: the FA, GL, GP, PK, PR, SP, saccharolipid (SL), and ST groups. It was found that the relative percentages of SP and GP in the skin lipids of the SDS population were significantly higher than those of the SOS and HS population. Meanwhile, the relative proportion of ST was lower in the SDS population than in the other two groups. In addition, GL and PR accounted for a higher proportion in the SOS population. However, the proportions of FA, PK, and SL remained relatively consistent across all three groups (Figure 3a).

### 3.5. Analysis of the Lipids in the Stratum Corneum of SDS, SOS, and HS

After analyzing the stratum corneum lipids of the three groups, a total of 38 lipid components were identified. These lipids were categorized into seven major groups: FA, GL, GP, PK, PR, SP, and ST. As shown in Figure 3b, in the SDS group, the relative percentages of SP and PR were significantly higher than those of in the SOS and HS groups. In contrast, the relative proportions of FA, GP, PK, and ST were lower in the SDS group than in the other two groups. However, the proportion of GL was similar across all three groups.

## 4. Discussion

Sensitive skin, especially SDS and SOS, exhibited differences in physiologic properties and lipid composition compared to HS, and these differences profoundly affected the integrity and stability of the skin barrier function. In SOS, hyperactivity of sebaceous glands led to excess oil production. This high oil content may have disturbed the microbial balance of the skin, which could lead to skin problems such as acne and seborrheic dermatitis [31,32]. This finding echoed the results of a previous study that analyzed lipidomics in patients with oily sensitive skin (OSS), both highlighting the role of abnormal oil production in the pathology of sensitive skin [12]. On the contrary, SDS faced the double challenge of low oil content and insufficient moisture in the stratum corneum, which not only resulted in dry, flaky skin, but also greatly weakened the skin barrier’s ability to defend itself [33,34]

In terms of the skin physiological parameters, this study revealed that both SOS and SDS types had higher TEWL values than healthy skin group. This was in agreement with the findings of Sun Suji et al. and further confirmed that skin barrier dysfunction was a major feature of sensitive skin [35,36]. Impaired barrier function and thinning of the stratum corneum in populations with sensitive skin led to an increased ability of exogenous substances to penetrate the skin, thus causing inflammatory skin reactions [5].

In terms of lipid composition, GL, GP, PK, ST, and FA were more abundant in HS than in SDS. These lipid components played an important role in maintaining skin health, elasticity, and moisturization. For example, GL and GP were pivotal in augmenting sebum fluidity and enhancing skin moisturization. GP, constituting the primary building blocks of cell membranes, safeguarded their structural integrity and stability, whereas GL facilitated the regulation of sebum lubricity [18]. The high contents of PK and ST correlated with their potent anti-inflammatory and immune-regulatory properties, contributing positively to skin wellness [19,20,21]. And FA were key players in maintaining normal skin barrier function. In particular, long-chain FA could reduce the loss of water from the skin surface, resist the entry of harmful substances, and maintain normal skin barrier function [22].

SP was a key component of intercellular lipids in the stratum corneum of the skin and was essential for maintaining skin barrier function. It was noteworthy that the relative content of SP, especially ceramides, was high in the SDS group. Ceramides, composed of sphingosine linked to fatty acids via amide bonds, accounted for approximately 45–50% of the total intercellular lipids [37]. Ceramides played a crucial role in building and maintaining the permeability barrier function of the skin [38]. As reported by Luczaj W et al., high ceramide content has been observed in the skin of psoriasis, which is usually thought to be abnormal in the process of epidermal differentiation [39]. Although SDS cannot be directly equated with psoriasis, further research is needed to determine whether there are similarities in the formation process. The relatively elevated ceramide content may have been a compensatory mechanism adopted by the skin to compensate for the deficiency of other lipid components. Under normal conditions, ceramides, cholesterol, and free fatty acids form an epidermal lipid barrier in the stratum corneum in equimolar proportions, effectively preventing TEWL and ionic penetration [40,41]. However, even with a high ceramide content, the overall function of the skin barrier might still have been impaired if other lipid components were insufficient. Therefore, the management and treatment of SDS should have focused on restoring the balance of lipid components to promote the overall restoration of skin barrier function. The role of SP, as crucial signaling molecules, must also be taken into account with respect to their generation, alterations, and influence on skin barrier formation during the skin differentiation process.

For SOS, no significant differences were observed with HS in the composition of both sebaceous gland lipids and stratum corneum intercellular lipids. This similarity may stem from the fundamental consistency of lipid composition and the existence of a universal, common physiological mechanism for the skin’s ability to maintain its barrier function, moisturize, and defend itself against external environmental aggressions [42]. The fact that SOS is similar to HS in terms of lipid composition may indicate that sensitivity is not caused by differences in the major components of sebum or intercellular lipids of the stratum corneum. Sensitivity may be related to the proportion or functional state of these lipid components, rather than just their presence or total amount. Therefore, an in-depth investigation of the dynamic interactions, structural properties, and biological activities of lipid molecules is essential for unraveling the pathomechanisms of sensitive skin and for developing targeted skincare strategies.

Significant disparities in lipid composition of the sebaceous glands and stratum corneum were observed between individuals with SOS and SDS. Those with SOS exhibited elevated levels of GL, GP, PK, ST, FA, and PR, aligning with the physiological attributes of sebum secretion. Notably, the high concentration of GP and GL likely contributed to the increased fluidity of sebum and augmented the skin’s moisturizing capacity [43]. Concurrently, the surge in FA analogs and ST may have indicated adaptive modifications in the skin’s barrier maintenance capabilities, potentially diminishing sensitivity. Moreover, these constituents might have played a role in mitigating inflammation and redness, prevalent symptoms in those with SOS [44]. PR played an important role in cell membrane composition, signaling, and cell recognition.

However, the current study has some limitations. Firstly, compared with Bi et al.’s UPLC-Q-TOF-MS comparison of lipid profiles of two age groups of dry-skinned women and Chen et al.’s analysis of surface lipids on the skin of the hands of healthy Chinese women of different ages [45,46], the study was limited in its representativeness because of the overly centralized distribution of the study population in terms of age and gender, making it difficult to comprehensively reflect the characteristics of the entire group. Secondly, the experiment was conducted during the winter season in northern China, where the outdoor temperature fluctuates at 10 ± 5 °C and the humidity is only 10–20%. In such an environment, the skin barrier function of the subjects may be more easily disturbed by external factors [47]. In addition, the increase in skin moisture content may have led to more water loss, which may have had some impact on the experimental results. Although we strictly controlled the experimental conditions, such as ensuring that subjects were not allowed to go out after entering the test environment, prohibiting the shading of the test site to minimize the effect of temperature and humidity changes on the experimental results, and requiring subjects and experimenters to remain quiet and sit in a sitting position to avoid the interference of air movement on the test site, the environmental factors may still have a certain impact on the accuracy of the results. Third, this study was mainly based on static data, which failed to reflect the dynamic changes in skin physiological indicators and lipid composition in different time periods and conditions. Fourth, the physiological parameters and lipid composition of the skin are affected by a variety of factors, including age, gender, genetics, diet, and lifestyle habits [29,48]. Although this study attempted to control for the variables of age and gender, individual differences may still have an impact on the results.

## 5. Conclusions

In this study, the facial physiological indicators, lipid composition, and distribution patterns of the SDS, SOS, and HS groups were analyzed in depth using physiological index tests and untargeted lipidomics techniques. In terms of physiological indicators, notable disparities emerged in the oil content and stratum corneum water content between the SOS and both the HS and SDS groups. TEWL values were found to be higher in the sensitive skin groups (SDS and SOS) than in the HS group, indicating possible skin barrier dysfunction. It was shown that the proportion of SP in stratum corneum lipids was significantly higher in the SDS group, whereas there was no significant difference in lipid composition between the SOS and HS groups. This result suggests that skin barrier dysfunction in the SDS group may be closely related to lipid (especially ceramide) metabolism disorders. This study provides a solid theoretical and scientific basis for skin health management and skin care product development. Future studies need to expand the samples, conduct longitudinal analyses, and deeply investigate the dynamic interactions, structural properties, and bioactivities of lipid molecules. Further lipidomics research will be essential for exploring the spatial distribution of SP in the deeper layers of the epidermis.

## Figures and Tables

**Table 1:** Differences in physiological indicators of SDS, SOS, and HS.

Physiological Indicators	SDS	SOS	HS	P		
				SDS HS	SOS HS	SDS SOS
Oil content (μg/cm^2^)	38.22 ± 26.18	105.23 ± 52.93	45.26 ± 24.38	0.468	<0.05	<0.05
Stratum corneum water content (a.u.)	40.10 ± 9.32	52.69 ± 12.65	57.16 ± 7.96	0.141	<0.05	<0.05
TEWL (g/m^2^ h)	10.88 ± 2.79	10.13 ± 2.28	9.26 ± 2.48	0.618	0.343	0.173

Physiological Indicators

SDS

SOS

HS

P

**Table 2:** Information on potential lipid markers of sebaceous glands in the SDS and HS groups.

Lipid Type	Description	Formula	M/Z	*p* Value	FC	Highest Mean
GP	PS (P-20:0/21:0)	C47H92NO9P	863.6881942	0.000	4.041	SDS
	PI (P-20:0/22:4(7Z,10Z,13Z,16Z))	C51H91O12P	944.6623105	0.000	3.374	
	OHOOA-PS	C32H56NO12P	678.3626075	0.000	3.362	
GL	TG (20:5(5Z,8Z,11Z,14Z,17Z)/22:6(4Z,7Z,10Z,13Z,16Z,19Z)/20:5(5Z,8Z,11Z,14Z,17Z)) (d5)	C65H89D5O6	998.729861	0.000	3.386	
PK	Protorifamycin I	C35H45NO10	622.2994686	0.000	3.839	
PR	Glucopyranosyl-1-O-(4,4′-diapo-7′,8′,11′,12′-tetrahydrolycopen-4-oate)-6-O-(2-methylbutanoate)	C41H60O8	698.4636448	0.038	15.493	
ST	1alpha,25-dihydroxy-2beta-(6-hydroxyhexyl) vitamin D3	C33H56O4	534.4499808	0.000	3.869	
	Estradiol-17beta 3-sulfate	C18H24O5S	353.1432017	0.000	3.311	
GP	PA (O-16:0/13:0)	C32H65O7P	610.4798065	0.006	11.392	HS
	PE (P-20:0/22:6(4Z,7Z,10Z,13Z,16Z,19Z))	C47H82NO7P	786.5796597	0.002	8.194	
	PI (P-16:0/20:4(5Z,8Z,11Z,14Z))	C45H79O12P	825.5259817	0.003	7.818	
	PC (O-16:0/0:0)	C24H52NO6P	499.3872606	0.002	7.449	
	PG (O-20:0/14:1(9Z))	C40H79O9P	752.5815589	0.006	7.131	
	PG (O-16:0/12:0)	C34H69O9P	653.4732924	0.006	6.24	
	PC (18:4(6Z,9Z,12Z,15Z)/20:1(11Z))	C46H82NO8P	830.5638364	0.007	3.72	
	PA (P-16:0/22:6(4Z,7Z,10Z,13Z,16Z,19Z))	C41H69O7P	687.4776526	0.000	3.629	
GL	1-O-(2R-hydroxy-tetradecyl)-sn-glycerol	C17H36O4	327.2505918	0.036	10.465	
	1-O-(2R-hydroxy-heneicosanyl)-sn-glycerol	C24H50O4	425.3594323	0.001	7.783	
	MGMG (16:3(7Z,10Z,13Z)/0:0)	C25H42O9	485.2773161	0.017	7.035	
	MGDG (18:3(9Z,12Z,15Z)/18:4(6Z,9Z,12Z,15Z))	C45H72O9	774.5488086	0.000	4.139	
	DG (12:0/16:1(9Z)/0:0) [iso2]	C31H58O5	493.4229286	0.000	4.068	
	1-O-(2R-hydroxy-docosanyl)-sn-glycerol	C25H52O4	439.3755923	0.035	3.824	
	1-O-(2R-hydroxy-eicosanyl)-sn-glycerol	C23H48O4	411.3449934	0.026	3.757	
	2-(8-[5]-ladderane-octanyl)-sn-glycerol	C23H38O3	385.2705996	0.003	3.419	
	DG (12:0/16:1(9Z)/0:0) [iso2]	C31H58O5	493.4229286	0.000	4.068	
	1-O-(2R-hydroxy-docosanyl)-sn-glycerol	C25H52O4	439.3755923	0.035	3.824	
	1-O-(2R-hydroxy-eicosanyl)-sn-glycerol	C23H48O4	411.3449934	0.026	3.757	
	2-(8-[5]-ladderane-octanyl)-sn-glycerol	C23H38O3	385.2705996	0.003	3.419	
	DG (14:0/16:1(9Z)/0:0) [iso2]	C33H62O5	521.4540388	0.000	3.369	
PK	Poinsettifolin B	C30H34O5	497.2314437	0.018	27.124	
	Uvaricin	C39H68O7	671.4831433	0.007	3.507	
SP	Cer (d18:1/27:0)	C45H89NO3	674.6836807	0.001	10.722	
	N,N,N-trimethyl-sphingosine	C21H44NO2+	360.3720076	0.008	3.897	
PR	Zeaxanthin sulfate	C40H56O5S	666.4216044	0.038	4.422	
	Ustusolate G	C21H28O7	415.1726074	0.000	4.294	
	Rhodoquinone-9	C53H81NO3	797.6572202	0.000	3.539	
ST	Limnantheoside C	C38H62O16	775.4124783	0.013	6.923	
	1alpha,2alpha:4alpha,5alpha-diepoxy-3alpha,6beta-dihydroxy-ergosta-7,24(28)-dien-6-one-21-oic acid	C28H40O6	490.3169262	0.000	6.343	
	19-oxodesacetylcinobufagin	C24H30O6	437.1944656	0.000	5.737	
	1beta,3beta,5alpha,6beta-tetrahydroxyandrostan-17-one	C19H30O5	337.2034079	0.001	5.335	
	Suberoretisteroid E	C29H44O7	527.2968084	0.000	4.221	
	Lamellosterol A	C27H48O7S	499.3110574	0.003	4.076	
	Fasciculic acid C	C38H63NO11	710.4474808	0.029	3.865	
	Vitamin D3 glucosiduronate	C33H52O7	583.3593119	0.000	3.452	
	Bufogarlide A	C24H26O4	379.192273	0.009	3.302	
FA	Oleoyl-EA (d2)	C20H37D2NO2	310.3084792	0.004	7.741	
	karalicin	C14H20O6	267.121575	0.000	7.708	
	(Z)-7-(5-((1E,3E,6Z,9Z)-dodeca-1,3,6,9-tetraen-1-yl)-1,2-dioxolan-3-yl)-7-hydroperoxyhept-4-enoic acid	C22H32O6	415.2071569	0.000	5.301	
	5-hydroperoxy-7-[3,5-epidioxy-2-(2-octenyl)-cyclopentyl]-6-heptenoic acid	C19H30O6	372.2395883	0.002	5.256	
	2E,4E-undecadien-8,10-diynoic acid phenethylamide	C19H19NO	553.2839167	0.008	4.162	
	Lanoceric acid	C30H60O4	507.4382958	0.000	4.03	
	Bromovulone I	C21H29BrO4	425.1328207	0.000	3.754	
	Erucamide	C22H43NO	360.3234737	0.000	3.644	
	O-(17-carboxyheptadecanoyl) carnitine	C25H47NO6	458.3453896	0.039	3.441	

**Table 3:** Information on potential lipid markers of sebaceous glands in the SDS and SOS.

Lipid Type	Description	Formula	M/Z	*p* Value	FC	Highest Mean
GP	PS (P-20:0/21:0)	C47H92NO9P	863.6882	0.000	2.954	SDS
	PI (O-20:0/20:0)	C49H97O12P	926.7039	0.000	2.336	
GL	TG (20:5(5Z,8Z,11Z,14Z,17Z)/22:6(4Z,7Z,10Z,13Z,16Z,19Z)/20:5(5Z,8Z,11Z,14Z,17Z)) (d5)	C65H89D5O6	998.7299	0.000	2.948	
	DG (19:0/0:0/19:0) (d5)	C41H75D5O5	658.6372	0.000	2.269	
SP	Cer (d18:1/28:0)	C46H91NO3	688.6937	0.005	6.736	
	Cer (m18:1(4E)/26:1(17Z))	C44H85NO2	660.6625	0.003	4.378	
	Cer (t18:0/27:0(2OH))	C45H91NO5	708.6876	0.000	3.732	
	Cer (t20:0/26:0)	C46H93NO4	706.704	0.003	3.424	
	Hypoculoside	C30H61NO8	586.4293	0.000	3.417	
	1-O-carboceroyl-Cer (d18:1/18:0)	C64H125NO4	994.9454	0.001	3.241	
	Cer(d18:1/29:0)	C47H93NO3	702.7119	0.031	3.196	
	Cer(d18:2/25:0)	C43H83NO3	662.6417	0.000	2.798	
	Cer(d18:2/15:0)	C33H63NO3	522.4865	0.000	2.617	
	Cer(d18:0/16:0)	C34H69NO3	540.5373	0.002	2.549	
	Cer(t18:0/21:0)	C39H79NO4	626.6078	0.000	2.297	
PR	Glucopyranosyl-1-O-(4,4′-diapo-7′,8′,11′,12′-tetrahydrolycopen-4-oate)-6-O-(2-methylbutanoate)	C41H60O8	698.4636	0.047	10.917	
	Menaquinone-9	C56H80O2	767.6089	0.000	2.578	
FA	N-(20-hydroxyarachidoyl)-5-hydroxytryptamine	C30H50N2O3	509.3724	0.000	4.233	
	ascr#11	C10H18O6	235.1183	0.005	2.831	
GP	PI(P-16:0/20:4(5Z,8Z,11Z,14Z))	C45H79O12P	825.526	0.005	8.912	SOS
	PG(O-16:0/12:0)	C34H69O9P	653.4733	0.009	5.681	
	PA(O-16:0/13:0)	C32H65O7P	610.4798	0.024	5.02	
	PE(P-20:0/22:6(4Z,7Z,10Z,13Z,16Z,19Z))	C47H82NO7P	786.5797	0.026	3.527	
	PC(18:4(6Z,9Z,12Z,15Z)/20:1(11Z))	C46H82NO8P	830.5638	0.045	3.463	
	PA(P-16:0/22:6(4Z,7Z,10Z,13Z,16Z,19Z))	C41H69O7P	687.4777	0.002	2.36	
GL	MGTS(18:0/0:0)	C28H55NO6	519.4373	0.000	5.414	
	DG(13:0/16:1(9Z)/0:0)[iso2]	C32H60O5	507.4397	0.000	4.129	
	MGDG(18:3(9Z,12Z,15Z)/18:4(6Z,9Z,12Z,15Z))	C45H72O9	774.5488	0.010	3.072	
	DG(12:0/16:1(9Z)/0:0)[iso2]	C31H58O5	493.4229	0.000	2.968	
	DG(O-16:0/18:1(9Z))	C37H72O4	603.5314	0.005	2.642	
	TG(22:5(7Z,10Z,13Z,16Z,19Z)/22:6(4Z,7Z,10Z,13Z,16Z,19Z)/22:6(4Z,7Z,10Z,13Z,16Z,19Z))[iso3]	C69H100O6	513.3835	0.043	2.48	
PK	Poinsettifolin B	C30H34O5	497.2314	0.008	14.435	
	catechin 3-dodecanoate	C27H36O7	495.235	0.002	2.833	
	5-((5Z,8Z,11Z,14Z)-heptadeca-5,8,11,14-tetraen-1-yl)resorcinol	C23H32O2	339.2318	0.001	2.799	
SP	Cer(d18:1/27:0)	C45H89NO3	674.6837	0.004	5.548	
	Rhizochalinin D	C35H70N2O8	647.5215	0.007	4.151	
	N,N,N-trimethyl-sphingosine	C21H44NO2+	360.372	0.006	3.882	
PR	Nonaflavuxanthin	C45H62O	601.4773	0.002	7.145	
	Rhodoquinone-9	C53H81NO3	797.6572	0.001	2.981	
	Ustusolate G	C21H28O7	415.1726	0.003	2.616	
	Glisoprenin B	C45H82O6	741.5997	0.001	2.582	
	2,2′-Diapocarotene-dial	C34H40O2	463.3014	0.001	2.344	
ST	19-oxodesacetylcinobufagin	C24H30O6	437.1945	0.020	3.551	
	1alpha,2alpha:4alpha,5alpha-diepoxy-3alpha,6beta-dihydroxy-ergosta-7,24(28)-dien-6-one-21-oic acid	C28H40O6	490.3169	0.019	3.155	
	Lamellosterol A	C27H48O7S	499.3111	0.008	2.648	
	Suberoretisteroid E	C29H44O7	527.2968	0.003	2.565	
FA	O-(17-carboxyheptadecanoyl)carnitine	C25H47NO6	458.3454	0.017	3.963	
	(Z)-7-(5-((1E,3E,6Z,9Z)-dodeca-1,3,6,9-tetraen-1-yl)-1,2-dioxolan-3-yl)-7-hydroperoxyhept-4-enoic acid	C22H32O6	415.2072	0.009	3.684	
	Lanoceric acid	C30H60O4	507.4383	0.000	2.794	
	Bromovulone I	C21H29BrO4	425.1328	0.010	2.274	

**Table 4:** Information on potential lipid markers in the stratum corneum for the SDS and HS groups.

Lipid Type	Description	Formula	M/Z	*p* Value	FC	Highest Mean
PK	Lasalocid A	C34H54O8	608.41604	0.000	2.171	HS
GL	TG(16:0/18:1(9Z)/20:2(11Z,14Z))[iso6]	C57H104O6	902.8153729	0.027	3.293	SDS
SP	Cer(d18:1/23:0)	C41H81NO3	618.6210692	0.004	6.737	
	Cer(d18:1/25:0)	C43H85NO3	646.6491518	0.001	4.458	
	Cer(t18:1(6OH)/23:0(2OH))	C41H81NO5	650.6071059	0.031	3.468	
	Cer(t18:0/23:0)	C41H83NO4	654.6418605	0.035	2.468	
FA	(21-Methyl-8Z-pentatriacontene	C36H72	522.5977113	0.024	3.519	

**Table 5:** Information on potential lipid markers in the stratum corneum for the SDS and SOS groups.

Lipid Type	Description	Formula	M/Z	*p* Value	FC	Highest Mean
SP	Cer (d18:1/23:0)	C41H81NO3	618.6210692	0.004	7.471	SDS
	Cer (t18:1(6OH)/23:0(2OH))	C41H81NO5	650.6071059	0.011	6.569	
	Cer (d18:1/25:0)	C43H85NO3	646.6491518	0.001	4.38	

## Data Availability

The raw data supporting the conclusions of this article will be made available by the authors on request.

## Supplementary Materials

The following supporting information can be downloaded at https://www.mdpi.com/article/10.3390/metabo15050292/s1: Table S1: Mobile Phase Gradient Conditions; Table S2: Mass spectrometry conditions; Figure S1: Differences in physiological indicators of SDS, SOS and HS; Table S3: Differences in skin physiological indicators of SDS, SOS and HS groups.
