# Supplementary material for: Utilizing Untargeted Lipidomics Technology to Elucidate Differences in Lipid Compositions Among Sensitive Dry, Sensitive Oily and Healthy Skin Types
Source: Metabolites. 2025 Apr 26;15(5):292. doi: 10.3390/metabo15050292 (PMC12113311; doi:10.3390/metabo15050292)
Supplement: Supplementary file 1 [file metabolites-15-00292-s001.zip › metabolites-3574224-supplementary.pdf]

Table S1 Mobile Phase Gradient Conditions

Time (min)	Flow rate (ml/min)	Mobile phase A (%)	Mobile phase B (%)
0.00	0.3	80	20
1.0	0.3	80	20
5.0	0.3	40	60
11.0	0.3	20	80
18.0	0.3	10	90
19.0	0.3	0.0	100.0
21.0	0.3	0.0	100.0
21.10	0.3	80	20
22.00	0.3	80	20

Table S2 Mass spectrometry conditions

Parameter	ESI+
Sample Temperature	10.0 °C
Column Temperature	50.0 °C
Analytical Model	sensitivity
Capillary	3.0 kV
Source Temperature	120 °C
Desolvation Temperature	500 °C
Tone Hole Gas Flow	50 L/h
Desolvation Gas Flow	900 L/h

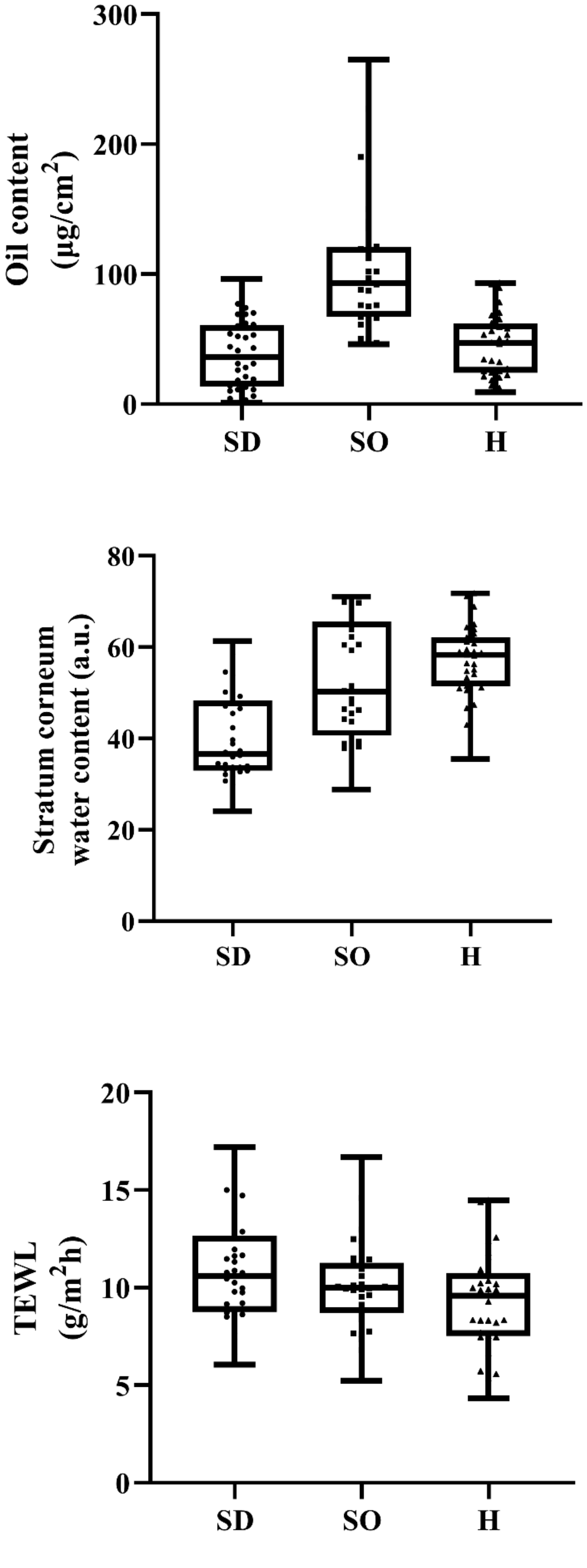

Figure S1 Differences in physiological indicators of SDS, SOS and HS. SD: sensitive dry; SO: sensitive oily; H: healthy.

Table S3 Differences in skin physiological indicators of SDS, SOS and HS groups.

group	Oil content ($\mu g/cm^2$)	Stratum corneum water content (a.u.)	TEWL (g/m^2 h)
H	53	58.10	12.56
H	47	54.00	14.37
H	89	58.27	14.48
H	56	56.20	9.90
H	27	58.70	8.19
H	12	62.93	10.34
H	92	50.93	9.27
H	25	55.03	7.52
H	33	54.73	9.88
H	93	62.07	7.67
H	34	71.77	5.36
H	21	58.93	6.63
H	58	59.43	11.56
H	62	64.33	9.86
H	20	64.97	5.55
H	48	58.97	5.70
H	50	68.83	9.97
H	32	61.83	12.39
H	68	71.13	8.32
H	70	51.40	11.79
H	78	61.17	7.46
H	18	42.93	10.21
H	59	63.87	4.33
H	46	51.07	8.32
H	25	46.60	10.91
H	24	60.77	10.18
H	14	53.27	8.31
H	53	47.30	9.59
H	9	56.40	8.30
H	65	35.47	10.75
H	22	50.53	7.46
SD	6	34.53	12.93
SD	53	36.33	11.62
SD	28	54.57	8.51
SD	74	36.97	6.05
SD	44	29.90	8.63
SD	69	58.50	11.65
SD	43	50.03	7.65
SD	41	32.70	13.78
SD	18	46.57	10.45

Table S3. Cont.

SD	21	32.10	17.20
SD	31	53.80	8.59
SD	10	32.87	9.97
SD	60	33.73	9.79
SD	52	61.30	9.20
SD	61	49.20	14.73
SD	31	48.67	9.15
SD	4	47.13	10.77
SD	54	35.97	10.88
SD	26	37.30	10.76
SD	1	50.17	8.87
SD	14	38.83	11.96
SD	11	33.97	6.58
SD	19	33.27	10.26
SD	69	34.3	14.78
SD	70	45.47	12.88
SD	11	42.37	8.73
SD	13	31.6	9.76
SD	51	39.67	7.34
SD	96	24.13	15.00
SD	62	30.73	11.48
SD	77	32.67	11.33
SD	3	33.73	16.80
SO	68	68.43	10.96
SO	75	70.90	9.60
SO	97	69.80	10.05
SO	121	37.80	9.92
SO	47	51.47	12.39
SO	119	37.40	14.59
SO	88	47.63	12.49
SO	67	50.23	9.97
SO	190	62.23	7.36
SO	87	28.80	8.69
SO	46	44.23	11.51
SO	102	63.93	5.24
SO	140	60.57	6.80
SO	63	48.63	9.53
SO	76	39.57	8.37
SO	227	40.63	11.45
SO	93	59.30	9.13
SO	66	38.20	12.98
SO	50	43.67	10.01

Table S3. Cont.

SO	102	46.40	10.11
SO	150	50.40	7.76
SO	135	46.23	10.06
SO	265	65.60	9.88
SO	188	60.50	7.60
SO	76	38.90	11.23
SO	92	71.03	9.94
SO	93	66.40	11.28
SO	116	69.93	7.66
SO	50	45.47	10.15
SO	61	39.27	10.59
SO	112	69.70	16.69
